# Pareidolia and Constipation in De Novo Parkinson’s Disease: Symptomatic Markers of a Shared Clinical Pattern

**DOI:** 10.7759/cureus.93833

**Published:** 2025-10-04

**Authors:** Takahide Wada, Junnosuke Ozawa, Kaoru Matsuoka, Naohito Ito, Hidetomo Murakami

**Affiliations:** 1 Department of Neurology, Showa University School of Medicine, Tokyo, JPN

**Keywords:** cognitive impairment and dementia, constipation, dementia, hallucinations, lewy body disease, pareidolia, parkinson’s disease

## Abstract

Background

Pareidolia, defined as the misperception of ambiguous stimuli as meaningful objects, is a frequent visual phenomenon observed in patients with Parkinson’s disease (PD). In contrast, constipation is one of the most prevalent non-motor symptoms in PD and appears early in the "body-first" subtype. Pareidolia shares partially overlapping pathophysiological mechanisms with visual hallucinations, which are more frequent in the body-first subtype of PD. The association between pareidolia and constipation, both of which can be observed in the early or prodromal state of PD, has not been previously explored. Therefore, in this study, we aimed to explore the potential relationship between pareidolia and constipation in drug-naïve patients with newly diagnosed PD who did not exhibit obvious hallucinations.

Methods

Thirty-two consecutive drug-naïve patients with PD admitted to Showa Medical University East Hospital between April 2022 and September 2024 were enrolled. Patients underwent comprehensive neurological and cognitive evaluations, including the Movement Disorder Society-Unified Parkinson’s Disease Rating Scale (MDS-UPDRS) Part III, Hoehn and Yahr stage (H-Y), Mini-Mental State Examination (MMSE), and Frontal Assessment Battery (FAB). Constipation was defined based on the Rome IV criteria. Pareidolia was assessed using the Noise Pareidolia Test (NPT).

Results

Among the 32 patients, 17 (53.1%) were pareidolia-positive and 26 (81.3%) had constipation. Constipation was significantly more common in the pareidolia-positive group than in the pareidolia-negative group (61.5% vs. 16.7%, p = 0.047). Patients with pareidolia were significantly older at disease onset and diagnosis (p = 0.002 and p = 0.004, respectively), had higher H-Y stages (p = 0.049), and lower FAB scores (p = 0.018). The MMSE total scores tended to be lower in the pareidolia-positive group (p = 0.053). However, compared with those without constipation, patients with constipation showed higher H-Y and MDS-UPDRS III scores (p = 0.012 and p = 0.047, respectively), and lower MMSE (p = 0.025) and FAB scores (p = 0.078).

Conclusion

Patients with pareidolia had more severe motor symptoms and cognitive impairment than those without, and the same pattern was observed when comparing patients with and without constipation. This suggests a potential association between pareidolia and constipation in PD, similar to the relationship between hallucinations and constipation. Our findings indicate that pareidolia and constipation may share a common pathological background, potentially reflecting the body-first subtype of PD.

## Introduction

Parkinson’s disease (PD) is a common neurodegenerative disorder characterized by not only parkinsonism but also various non-motor symptoms such as cognitive impairment, psychiatric symptoms, autonomic dysfunction, and rapid eye movement sleep behavior disorder (RBD) [1]. Psychiatric symptoms in PD include hallucinations, depression, anxiety, apathy, psychosis, and impulse control [2]. Furthermore, minor hallucinations are characteristic features observed in Lewy body diseases, including PD and dementia with Lewy bodies (DLB) [3,4]. Minor hallucinations, including illusions, sense of presence, and passage hallucinations, are not only relevant for the diagnosis of PD but are also associated with patients’ quality of life and clinical outcomes [3-5].

Pareidolia is a type of illusion in PD in which real objects are misidentified, and it differs from visual hallucinations, which involve perceiving objects that do not exist [6]. Pareidolia may involve dysfunction of the primary visual cortex and visual association areas in the occipital lobe, as well as the frontal lobe, and because it shares common pathophysiological mechanisms with hallucinations, patients with PD and pareidolia have an increased risk of developing hallucinations [7,8]. Furthermore, patients with idiopathic RBD, which is thought to involve dysfunction of brainstem structures such as the dorsal raphe nucleus and locus coeruleus, have been reported to exhibit pareidolia more frequently and have a higher risk of converting to Lewy body disease, a well-established prodromal marker of Lewy body disease [9]. In contrast, although pareidolia has been reported to be associated with important non-motor symptoms in PD, including visual hallucinations and RBD, the clinical relationship between pareidolia and constipation remains unclear.

Constipation in PD has been associated with an increased prevalence of various non-motor symptoms, including olfactory dysfunction, urinary disturbances, apathy, sleep disorders, and pain [10]. Recent studies suggest that prodromal constipation may indicate a specific pathological subtype of PD, known as the “body-first” type [11]. In this subtype, it is hypothesized that α-synuclein pathology begins in the enteric nervous system or peripheral autonomic nerves, and ascends through the brainstem, passing through non-dopaminergic nuclei such as the locus coeruleus and raphe nuclei, before reaching the substantia nigra [12,13]. Constipation and other autonomic dysfunctions in PD have been linked to a more severe clinical subtype [11,12]. Evidence indicates that PD patients with constipation are more likely to develop non-motor symptoms such as cognitive impairment, hallucinations, and RBD, supporting the concept of the body-first subtype in which these features are particularly prominent [11,14]. Therefore, the presence of constipation in early-stage PD may serve as a potential marker indicating the pattern of coexisting non-motor symptoms.

Although both pareidolia and constipation are common non-motor symptoms in PD, their relationship has not been well explored. There may be overlapping regions of pathological involvement in both pareidolia and constipation, suggesting that they could share common underlying mechanisms. Therefore, in this study, we conducted an exploratory analysis to examine whether constipation is associated with pareidolia in drug-naïve patients with newly diagnosed PD who did not exhibit obvious hallucinations.

## Materials and methods

Patients

From April 2022 to September 2024, 32 patients were admitted to the Department of Neurology at Showa Medical University East Hospital for evaluation and were diagnosed with PD. PD diagnoses followed the Movement Disorder Society (MDS) Clinical Diagnostic Criteria and included both clinically established and probable PD [14]. All patients were drug-naïve at the time of diagnosis and were eligible if they could complete the full assessment battery during admission.

Clinical data

All the patients received a neurological examination by at least two neurologists. Their clinical data, including sex, age at onset, and age at diagnosis, were extracted from medical records. Motor symptoms were assessed using the Movement Disorder Society-Unified Parkinson’s Disease Rating Scale (MDS-UPDRS) Part III [15] and Hoehn and Yahr stage (H-Y) [16]. Cognitive function was evaluated using the Mini-Mental State Examination (MMSE) [17] and the Frontal Assessment Battery (FAB) [18]. Pareidolia was assessed using the Noise Pareidolia Test (NPT), and patients were considered pareidolia-positive if they reported one or more pareidolic responses [19]. The presence of constipation was defined according to the Rome IV criterion of constipation [20]. The MDS-UPDRS Part III was administered with permission. Assessments were conducted by treating clinicians and were not blinded to clinical information. All instruments were administered according to standardized procedures. There were no missing data for the variables included in the analyses; all analyses were conducted on complete cases. The Japanese version used in this study was licensed by the International Parkinson and Movement Disorder Society (MDS). Cognitive screening was performed using the Mini-Mental State Examination-Japanese (MMSE-J; Nihon Bunka Kagaku-sha Co., Ltd., Tokyo, Japan), produced under license from the original publisher, Psychological Assessment Resources, Inc. (Lake Magdalene, FL).

NPT

The NPT, developed at Tohoku University in Japan, is a brief neuropsychological assessment designed to elicit and quantify visual misperceptions [19]. During the test, 40 black-and-white images are sequentially presented, each for up to 30 seconds. Of these, eight images contain embedded human faces, while the remaining 32 consist of visual noise only. Participants are instructed to indicate whether or not they perceive a face in each image. If a face is perceived, they are asked to respond “yes” and identify its location; otherwise, they respond “no.” Prior to testing, participants are trained with clear instructions and three practice trials, and are told to respond “yes” only when a face is clearly visible. Participant responses are classified into three categories: “correct responses” include accurate identification of faces in target images or correct rejection of noise-only images, “pareidolic responses” include false identification of faces in noise-only images, and “misses” include failure to detect actual faces in face-containing images. No feedback is provided during the task, regardless of response accuracy. Patients were considered pareidolia-positive if they produced one or more pareidolic responses [21].

Constipation: Rome IV diagnostic criteria

Constipation was defined based on the Rome IV diagnostic criteria [20] for functional constipation. According to these criteria, patients were required to have experienced two or more of the following symptoms for at least three months, with symptom onset at least six months prior to diagnosis: straining during more than 25% of defecations, lumpy or hard stools in more than 25% of defecations, sensation of incomplete evacuation in more than 25% of defecations, sensation of anorectal obstruction/blockage in more than 25% of defecations, manual maneuvers to facilitate more than 25% of defecations, and fewer than three spontaneous bowel movements per week. In addition, patients who were taking laxatives for constipation at the time of assessment were also considered to have constipation.

Standard protocol approval, registration, and patient consent

In this cross-sectional study, we retrospectively used clinical evaluations and other background information obtained in routine clinical practice at Showa Medical University Hospital or Showa Medical University East Hospital from April 1, 2022, to September 30, 2024. The study was approved by the Showa University Research Ethics Review Board (approval number: 2024-214-A) and conducted in accordance with the principles outlined in the Declaration of Helsinki (as revised in 2013). Written informed consent was not obtained because the approved protocol for the study was based on the opt-out method, according to the Ethical Guidelines for Medical and Biological Research Involving Human Subjects (revised March 2023) of the Japanese Ministry of Education, Culture, Sports, Science and Technology, the Ministry of Health, Labor and Welfare, and the Ministry of Economy, Trade and Industry. Based on the protocol, the research plan was announced on the university's website and in advertisements from November 2023. Patients who did not want to participate were able to decline participation based on the opt-out method. Data were used for target patients who did not decline participation until the end of October 2024. Consequently, no patients whose clinical information was obtained for the study refused participation.

Statistical analysis

Continuous variables were assessed for normality and homogeneity of variance using the Shapiro-Wilk and Levene’s tests. Normally distributed data were analyzed using the Student’s t-test, while non-normally distributed variables were analyzed using the Mann-Whitney U test. Contingency analysis was performed using the chi-squared test, and multivariate analyses were performed using logistic regression. All statistical analyses were performed using SPSS Statistics version 28.0 (IBM Corp., Armonk, NY), and all tests were two-sided. A p-value of less than 0.05 was considered statistically significant, and a p-value of less than 0.1 was considered a trend toward significance.

## Results

Patient characteristics

Among the 32 patients, 20 met the criteria for clinically established PD, and 12 for clinically probable PD. A total of 17 patients exhibited pareidolia at the time of diagnosis, and 26 had constipation. The mean age at diagnosis was 71.8 ± 9.80 years, the UPDRS Part III score was 28.4 ± 13.6, and the H-Y stage was 2.46 ± 0.82. All patients showed improvement in motor symptoms after the initiation of dopaminergic therapy. None of the patients exhibited psychiatric symptoms, cognitive impairments, or obvious hallucinations suggestive of DLB [22].

Pareidolia

Among the 32 patients, 17 were classified as pareidolia-positive (Table 1). Constipation was more frequently observed in the pareidolia-positive group than in the pareidolia-negative group (p = 0.047). Compared with the pareidolia-negative group, the pareidolia-positive group was significantly older at both disease onset and diagnosis (p = 0.002 and p = 0.004, respectively). Patients with pareidolia also showed significantly higher H-Y stages (p = 0.049) and lower FAB (p = 0.018) and MMSE scores (p = 0.053). Although the univariate analysis indicated these associations, the multivariate logistic regression did not identify any independent predictors of pareidolia, including constipation, onset age, H-Y stage, and FAB score (Table 2).

**Table 1:** Univariate comparison of clinical characteristics between patients with PD with and without pareidolia. Data are expressed as means ± SDs. The MDS-UPDRS Part III was administered with permission. The MMSE-J was used under license from Psychological Assessment Resources, Inc. Japanese edition is published by Nihon Bunka Kagaku-sha Co., Ltd. (Tokyo, Japan). * P < 0.05 was considered statistically significant, and † p < 0.1 was considered a trend toward significance. Test statistics are reported as chi-square values (χ², df) for categorical variables and Mann–Whitney U values for non-parametric comparisons. SD: standard deviation; FAB: Frontal Assessment Battery; H-Y: Hoehn and Yahr stage; MMSE: Mini-Mental State Examination; PD: Parkinson’s disease; MDS-UPDRS: Movement Disorder Society-sponsored revision of the Unified Parkinson’s Disease Rating Scale.

	Total	Pareidolia (+)	Pareidolia (–)	p-value	Test statistic
Number, n	32	17	15		
Sex (male/female), n	18/14	10/7	8/7	0.755	χ² = 0.098, df = 1
Age at onset, years	70.6 ± 9.66	75.3 ± 6.93	65.2 ± 10.0	0.002*	U = 207.0
Age at diagnosis, years	71.8 ± 9.80	76.5 ± 6.79	66.5 ± 10.4	0.004*	U = 202.0
Diagnostic criteria established/probable, n	20/12	11/6	9/6	0.784	χ² = 0.075, df = 1
Tremor/rigidity onset, n	10/22	6/11	4/11	0.599	χ² = 0.276, df = 1
Constipation (+/-), n	26/6	16/1	10/5	0.047*	χ² = 3.942, df = 1
Disease duration, months	15.5 ± 12.0	15.1 ± 10.7	15.9 ± 14.1	0.970	U = 126.0
MDS-UPDRS Part Ⅲ [16]	28.4 ± 13.6	31.8 ± 13.1	24.4 ± 14.1	0.134	U = 173.0
H-Y [17]	2.46 ± 0.82	2.76 ± 0.75	2.13 ± 0.83	0.049*	U = 180.0
MMSE [18]	27.3 ± 2.35	26.5 ± 2.47	28.2 ± 2.04	0.053^†^	U = 76.5
FAB [19]	14.5 ± 3.07	14.1 ± 3.15	16.5 ± 2.25	0.018*	U = 65.0

**Table 2:** Factors associated with pareidolia: a multivariate logistic regression model. CI: confidence interval; FAB: Frontal Assessment Battery; H-Y: Hoehn and Yahr.

Factor	Odds ratio	p-value	95% CI
Age of disease onset	1.102	0.111	0.978 – 1.242
Presence of constipation	3.111	0.404	0.217 – 44.672
H-Y [17]	1.821	0.383	0.474 – 6.996
FAB [19]	0.512	0.885	0.613 – 1.276

Constipation

In the constipation group, there were no significant differences between the constipation and non-constipation groups in terms of age, sex, and disease duration (Table 3). However, patients with constipation had significantly higher scores on the MDS-UPDRS Part III (p = 0.047) and H-Y stage (p = 0.012), and significantly lower MMSE (p = 0.025) and FAB scores (p = 0.078).

**Table 3:** Univariate comparison of clinical characteristics between patients with PD with and without constipation. Data are expressed as means ± SDs. The MDS-UPDRS Part III was administered with permission. The MMSE-J was used under license from Psychological Assessment Resources, Inc. Japanese edition is published by Nihon Bunka Kagaku-sha Co., Ltd. (Tokyo, Japan). * P < 0.05 was considered statistically significant, and † p < 0.1 was considered a trend toward significance. Test statistics are reported as chi-square values (χ², df) for categorical variables and Mann–Whitney U values for non-parametric comparisons. SD: standard deviation; FAB: Frontal Assessment Battery; H-Y: Hoehn and Yahr stage; MMSE: Mini-Mental State Examination; PD: Parkinson’s disease; MDS-UPDRS: Movement Disorder Society-sponsored revision of the Unified Parkinson’s Disease Rating Scale.

	Constipation (+)	Constipation (–)	p-value	Test statistic
Number, n	26	6		
Sex (male/female), n	14/12	4/2	0.568	χ² = 0.326, df = 1
Age at onset, years	71.8 ± 9.43	65.0 ± 10.3	0.107	U = 112.0
Age at diagnosis, years	73.0 ± 9.55	66.6 ± 10.8	0.158	U = 108.0
Diagnosis criteria established /probable, n	16/10	4/2	0.815	χ² = 0.055, df = 1
Tremor/rigidity onset, n	19/7	3/3	0.272	χ² = 1.208, df = 1
Disease duration, months	14.5 ± 10.6	19.8 ± 18.3	0.524	U = 64.0
MDS-UPDRS Part Ⅲ [16]	30.7 ± 13.9	18.3 ± 8.21	0.047*	U = 124.5
H-Y [17]	2.65 ± 0.79	1.67 ± 0.51	0.012*	U = 129.0
MMSE [18]	26.9 ± 2.41	29.1 ± 1.16	0.025*	U = 32.0
FAB [19]	14.12 ± 3.15	16.50 ± 2.25	0.078^†^	U = 41.0

## Discussion

In this study, we evaluated pareidolia in drug-naïve patients with PD by classifying them based on the presence or absence of constipation. We also classified patients according to the presence of pareidolia and compared their clinical characteristics, including constipation status and cognitive function. We found that patients with PD and pareidolia were more likely to exhibit constipation, suggesting a possible association between pareidolia and constipation. Furthermore, patients with pareidolia had more severe motor symptoms and cognitive impairment than those without, and the same pattern was observed when comparing patients with and without constipation.

In the present study, patients with pareidolia or constipation exhibited more severe motor symptoms and cognitive impairment. Constipation in PD has been linked to visual hallucinations, severe motor symptoms, and cognitive decline, suggesting a subtype with widespread pathology [11,12,23]. Although pareidolia is defined as distinct from hallucinations, pareidolia partially overlaps with hallucinations in pathological features [3,6]. In the present study, among PD patients without obvious hallucinations, those with pareidolia and constipation tended to present with more severe motor symptoms and cognitive impairment, suggesting a specific subtype. Similar to the association between hallucinations and constipation, the evaluation of pareidolia prior to the onset of overt hallucinations may help identify this subtype at an earlier stage. Proving that the clinical subtype presenting with pareidolia and constipation shares common pathological degeneration will require additional imaging analyses extending beyond this study.

The body-first or brain-first hypothesis of PD is a disease model of the α-synuclein origin site and connectome model [11]. The brain-first subtype posits that the pathology starts unilaterally in the olfactory bulb or amygdala, whereas the body-first subtype posits that the pathology starts in the peripheral nervous system, most likely the enteric nervous system. Subsequently, it spreads via autonomic neurons to the sympathetic trunk and the brainstem, and from there invades the rest of the brain in a bottom-up ascending manner [11]. Compared with the brain-first subtype, the body-first subtype is characterized by more severe motor and cognitive impairment, a higher frequency of RBD strongly associated with pareidolia, and the early occurrence of constipation [9-11,24]. In the present study, patients with PD who exhibited pareidolia and those with constipation both showed more severe motor and cognitive impairment, suggesting that pareidolia and constipation may represent common non-motor features of the body-first subtype.

Constipation in PD is influenced by gut microbiota dysbiosis, which has been linked to cognitive impairment and mood disorders [25]. Previous studies have reported that patients with PD and constipation exhibit a higher prevalence of cognitive dysfunction [26]. Since pareidolia has also been associated with impaired executive function and attentional deficits [7,8], it is possible that constipation and pareidolia are interconnected through shared mechanisms involving cognitive impairment. In the present study, patients with PD and pareidolia and those with PD and constipation each showed lower FAB and MMSE scores compared with patients without pareidolia or constipation, respectively. These findings support the possibility that pareidolia and constipation are interrelated through shared mechanisms involving cognitive impairment.

This study has several limitations. First, it was a single-center, exploratory study with a relatively small sample size, and all participants were Japanese. We did not perform an a priori sample size (power) calculation to justify the enrollment of 32 patients, nor did we conduct a post hoc power analysis; therefore, the study may have been underpowered. Second, multiple statistical tests were conducted without a formal correction for multiple comparisons, so type I error cannot be excluded. Notably, variables identified as risk factors in univariate analyses, including constipation, did not remain independent predictors of pareidolia in the multivariable model, which may reflect limited power; age at onset is a particularly important confounder when considering the relationship between pareidolia and constipation. In PD, the presence of pareidolia has been associated with higher age and poorer cognitive function [27]. However, in the present multivariate analysis, age was not significantly associated with the presence of pareidolia, and therefore cannot be considered a confounding factor that negates the observed relationship between pareidolia and constipation. Third, inter-rater reliability for pareidolia assessment was not evaluated, which may limit the robustness of this measure. Furthermore, assessments were not blinded, which may introduce observer bias despite the use of standardized instruments and predefined criteria. Fourth, the temporal alignment between constipation history and pareidolia testing was not explicitly defined, and the cross-sectional design precludes conclusions about directionality or causality. Fifth, we included both clinically established and probable PD according to the MDS Clinical Diagnostic Criteria and did not perform stratified analyses by diagnostic certainty. This heterogeneity may limit the interpretability and generalizability of our findings. Finally, our interpretation based on the body-first versus brain-first hypothesis relied on the presence or absence of constipation at diagnosis; pathological validation (e.g., skin or colonic biopsies) would be required to substantiate this classification. Moreover, these subtypes are unlikely to apply to all patients with PD [28]. Nevertheless, this hypothesis provides a useful disease model for understanding PD heterogeneity and may help contextualize the observed association between pareidolia and constipation.

## Conclusions

We found a potential association between pareidolia and constipation in PD. These observations suggest that both pareidolia and constipation may share a common pathological background, possibly reflecting the body-first subtype of PD. We believe that this study will contribute to a deeper understanding of PD heterogeneity and the clinical significance of early non-motor features. Future large-scale multicenter studies with pathological investigations may be warranted to further validate our findings.
